# Group Analysis in MNE-Python of Evoked Responses from a Tactile Stimulation Paradigm: A Pipeline for Reproducibility at Every Step of Processing, Going from Individual Sensor Space Representations to an across-Group Source Space Representation

**DOI:** 10.3389/fnins.2018.00006

**Published:** 2018-01-22

**Authors:** Lau M. Andersen

**Affiliations:** NatMEG, Department of Clinical Neuroscience, Karolinska Institutet, Stockholm, Sweden

**Keywords:** MEG, analysis pipeline, MNE-Python, minimum norm estimate (MNE), tactile expectations, group analysis, good practice

## Abstract

An important aim of an analysis pipeline for magnetoencephalographic data is that it allows for the researcher spending maximal effort on making the statistical comparisons that will answer the questions of the researcher, while in turn spending minimal effort on the intricacies and machinery of the pipeline. I here present a set of functions and scripts that allow for setting up a clear, reproducible structure for separating raw and processed data into folders and files such that minimal effort can be spend on: (1) double-checking that the right input goes into the right functions; (2) making sure that output and intermediate steps can be accessed meaningfully; (3) applying operations efficiently across groups of subjects; (4) re-processing data if changes to any intermediate step are desirable. Applying the scripts requires only general knowledge about the Python language. The data analyses are neural responses to tactile stimulations of the right index finger in a group of 20 healthy participants acquired from an Elekta Neuromag System. Two analyses are presented: going from individual sensor space representations to, respectively, an across-group sensor space representation and an across-group source space representation. The processing steps covered for the first analysis are filtering the raw data, finding events of interest in the data, epoching data, finding and removing independent components related to eye blinks and heart beats, calculating participants' individual evoked responses by averaging over epoched data and calculating a grand average sensor space representation over participants. The second analysis starts from the participants' individual evoked responses and covers: estimating noise covariance, creating a forward model, creating an inverse operator, estimating distributed source activity on the cortical surface using a minimum norm procedure, morphing those estimates onto a common cortical template and calculating the patterns of activity that are statistically different from baseline. To estimate source activity, processing of the anatomy of subjects based on magnetic resonance imaging is necessary. The necessary steps are covered here: importing magnetic resonance images, segmenting the brain, estimating boundaries between different tissue layers, making fine-resolution scalp surfaces for facilitating co-registration, creating source spaces and creating volume conductors for each subject.

## Introduction

Magnetoencephalography (MEG) studies often include questions about how different experimental factors relate to brain activity. To test experimental factors, one can create contrasting conditions to single out the unique contributions of each experimental factor. Single subject studies using MEG would face two limitations in singling out the contributions of experimental factors. Firstly, the MEG signals of interest are mostly too weak to find due to the noise always present in MEG data, and secondly there is often an interest in making an inference from one's data to the population as a whole. Group level analyses can circumvent these limitations by increasing the signal-to-noise ratio and by allowing for an inference to the population as a whole. It should be mentioned though that single subject analyses can be meaningful for clinicians trying to diagnose patients. Epilepsy investigations are routinely carried out on single subjects. Despite the fact that most studies rely on group level comparisons to increase the signal-to-noise ratio and for allowing for inferences to the population, almost all tutorials are based on single subject analyses. In the current paper, part of a special issue devoted to group analysis pipelines, I try to remedy this for anyone fancying using the MNE-Python (Gramfort et al., 2013) analysis package. The example analysis that will be used is focused on group level source reconstruction analyses of evoked responses, since this is a very common strategy in the MEG literature. As such, the focus is on how to organize a data analysis pipeline, but for more general introductory information about MEG and the analysis of evoked fields in general, see Hämäläinen et al. (1993) and Hari and Puce (2017). The organizational principle will be that all parts, both within-subject and between-subject parts, of the analysis will be accessible from the Python interface using a single script. The data is structured according to the Magnetoencephalography Brain Imaging Data structure (MEG-BIDS) format to ease access to the data (Galan et al., 2017).

The basic idea of the current group pipeline is to set up a structure that allows for:

Dividing output files into folders belonging to the respective subjects and recordings.
Applying an operation across a group of subjects.
(Re)starting the analysis at any intermediate point by saving output for each intermediate point.
Plotting the results in a way that allows for changing the figures in a principled, but flexible manner.

A structure that allows for all four points will minimize the time that researchers have to spend on (1) double-checking that the right input goes into the right functions; (2) making sure that output and intermediate steps can be accessed meaningfully; (3) applying operations efficiently across groups of subjects; (4) re-processing data if changes to any intermediate step are desirable.

## The neuroscientific experiment

Since the focus is on how to conduct a group analysis, the neuroscientific questions answered with the pipeline are neither novel nor interesting. The focus is rather on the pipeline, which can facilitate other experimenters' research, so that they efficiently can answer their own novel and interesting questions. The reserved digital object identifier (DOI) for the data repository, where data for this experiment and scripts for the pipeline can be freely downloaded is: 10.5281/zenodo.998518. The corresponding URL is: https://zenodo.org/record/998518. The study that the data are taken from is not published yet. The updated and maintained github code can be found at https://github.com/ualsbombe/omission_frontiers.

### Goal of analysis

**Figure 1:**
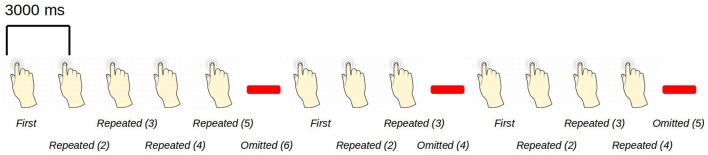
An example sequence of the experimental paradigm is shown. The annotations on the bottom show the coding used throughout for the different events of interest. Stimulations happened at a regular pace, every three seconds. When omissions occurred, there were thus six seconds between two consecutive stimulations.

**Figure 2:**
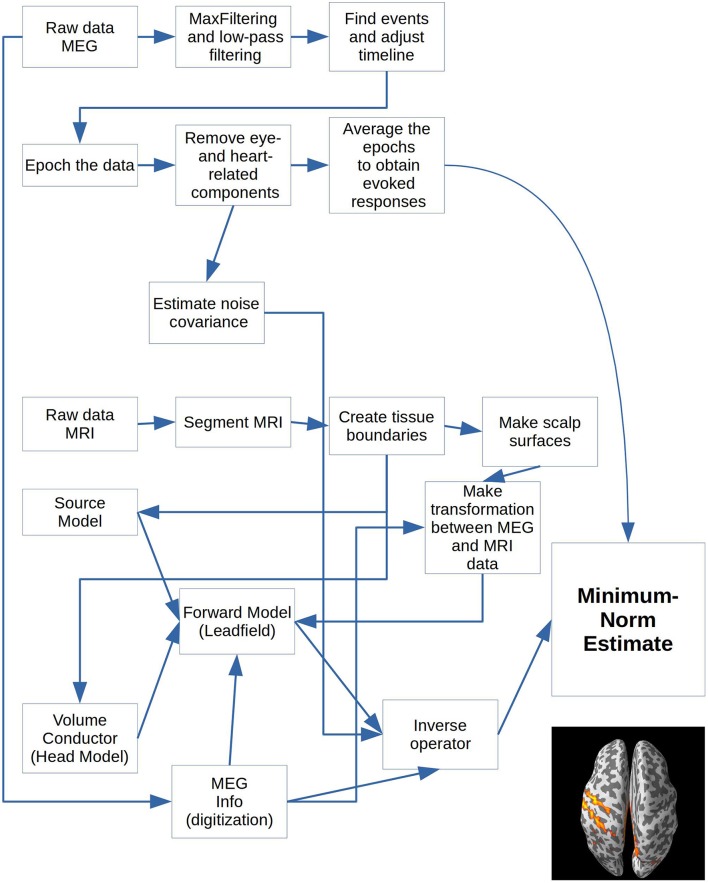
Cookbook for performing the Minimum-Norm Estimates for a single subject.

The goal of the analysis is to make a statistical appraisal of the neural activation evoked from the stimulation of the right index finger. The question is whether evidence can be found against the null hypothesis that neural activation in the contralateral somatosensory cortex does not depend on whether or not the right index finger is stimulated. This has been shown to be a robust effect, which makes it suitable for illustrating the pipeline. In reality, it is well known that stimulation of the finger evokes (at least) two evoked responses, the first after ~60 ms and the second after ~135 ms (Hari et al., 1984). The first localizes to contralateral primary somatosensory cortex and the second to bilateral secondary somatosensory cortex. To meet this goal, the following are sufficient: (1) evoked responses from each subject's raw data. (2) volume conductors and forward models based on the subjects' magnetic resonance images (MRIs) of their brains. (3) minimum norm estimates for each subject (4) statistics across the events based on the individual source reconstructions. The paradigm (Figure 1) and the whole analysis pipeline for each subject is shown in Figure 2.

A far from comprehensive list of studies facilitating similar pipelines includes: word recognition paradigms (Halgren et al., 2002; Pulvermüller et al., 2003); language lateralization assessment (Raghavan et al., 2017); auditory stimulation (Coffey et al., 2016) expectations toward painful stimulation (Fardo et al., 2017); face processing (Junghöfer et al., 2017); cross-sensory activations in visual and auditory cortices (Raij et al., 2010); somatosensory response activations (Nakamura et al., 1998) and many more.

### Subjects

Twenty participants volunteered to take part in the experiment (eight males, twelve females, Mean Age: 28.7 y; Minimum Age: 21; Maximum Age: 47). The experiment was approved by the local ethics committee, Regionala etikprövningsnämnden i Stockholm. Both written and oral informed consent were obtained from all subjects.

### Paradigm

The paradigm is based on building up tactile expectations by rhythmic tactile stimulations. These tactile expectations are every now and then violated by omitting otherwise expected stimulations (Figure 1). The inter-stimulus interval was 3,000 ms. Around every twenty-five trials, and always starting after an omission, periods of non-stimulation occurred that would last 15 s. The first six seconds worked as a wash-out period, and the remaining nine seconds were cut into three epochs of non-stimulation. There are thus nine trigger values in the data responding to nine different kinds of events (Table 1).

**Table 1:** Mapping of trigger values and annotated events.

**Trigger value**	**Annotation**	**Notes**	**Number of trials**
1	*Standard 1*	First stimulation	~200
2	*Standard 2*	Second stimulation	~200
3	*Standard 3*	Third stimulation	~200
4	*Standard 4*	Fourth stimulation	~135
5	*Standard 5*	Fifth stimulation	~66
13	*Omission 4*	Omission following third stimulation	~66
14	*Omission 5*	Omission following fourth stimulation	~66
15	*Omission 6*	Omission following fifth stimulation	~66
21	*Non-Stimulation*	Absence of stimulation outside the rhythmic stimulation sequences	~130

During the stimulation procedure, participants were watching an unrelated nature programme with sound being fed through sound tubes into the ears of participants at ~65 dB, rendering the tactile stimulation completely inaudible. Participants were instructed to pay full attention to the movie and no attention to the stimulation of their finger. In this way, expectations should be mainly stimulus driven, and thus not cognitively driven or attention driven.

An analysis of evoked responses will be carried out. The specific parameters going into the analysis will become apparent in the analysis steps below.

### Preparation of subjects

In preparation for the MEG-measurement each subject had their head shape digitized using a Polhemus Fastrak. Three fiducial points, the nasion and the left and right pre-auricular points, were digitized along with the positions of four head-position indicator coils (HPI-coils). Furthermore, about 200 extra points, digitizing the head shape of each subject, were acquired.

### Acquisition of data

Data was sampled on an Elekta TRIUX system at a sampling frequency of 1,000 Hz and on-line low-pass and high-pass filtered at 330 and 0.1 Hz, respectively. The data were first MaxFiltered (-v2.2) (Taulu and Simola, 2006), movement corrected and line-band filtered (50 Hz). MaxFiltering was done with setting the coordinate frame to the head coordinates, setting the origin of the head to (0, 0, 40 mm), setting the order of the inside expansion to 8, setting the order of the outside expansion to 3, enabling automatic detection of bad channels and doing a temporal Signal Space Separation (tSSS) with a buffer length of 10 s and a correlation limit of 0.980. Calibration adjustment and cross-talk corrections were based on the most recent calibration adjustment and cross-talk correction performed by the certified Elekta engineers maintaining the system.

### Conventions

<variable> will be used to refer to the variable called “variable.”
***function*** will be used to refer to the function called “function.”
[parameter] will be used to the parameter called “parameter.”
*script* will be used to refer to the script called “script.”

### Requirements

The packages in Table 2 are required to run the scripts, and the versions listed are the ones that have been used to test the scripts.

**Table 2:** Packages, their purposes and origins, that are necessary for the pipeline.

**Packages**	**Purposes**	**Origin**	**Version**
Mne	Analysing MEG data	Anaconda	0.15
Numpy	Easing numerical operations	Anaconda	1.9.2
Os	Interacting with operating system	Anaconda	From python 2.7.11
Matplotlib	Enabling MATLAB-like plotting	Anaconda	1.4.3
Scipy	Getting statistical functions and distributions	Anaconda	0.15.1
Mayavi	Plotting 3D-plots	Anaconda	4.4.0

## Code

### General structure of the code

The idea behind this pipeline is that each processing step can be run independently of what is in the workspace of the python interpreter as long as the appropriate processing step has been applied once earlier. To ascertain this almost all the functions begin with loading the appropriate data and by saving the processed data.

MNE-Python functions are used to do the actual operations. The functions supplied in this pipeline mostly serve as convenience functions that load the right data, process it and finally save it so it can be loaded for the next processing step.

#### Structure of *pipeline.py*

This is the main script, which is used to designate which operations should be run on the MEG data. The pipeline script is ordered into five blocks of code: Imports (Code Snippet 1), Paths (Code Snippet 2), Operations (Code Snippet 3), Parameters (Code Snippet 4), and the Processing Loop. It can be found one directory up from <script_path> (Code Snippet 1).

##### Imports

This sets the home folder <home_path>, which should to be changed to the user's home folder and imports necessary packages. Also make sure that the path to the scripts <script_path> points to the appropriate path where the below scripts can be found (Code Snippet 1). Finally also set the project name <project_name> to the folder where your analysis is stored.

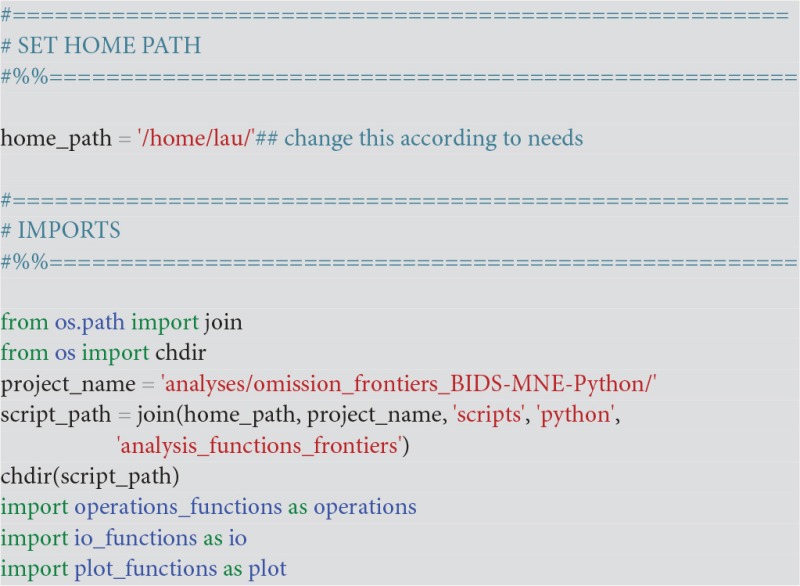



**Code Snippet 3**. Importing packages necessary for the pipeline.

##### Input/output—io_functions.py

The file *io_functions.py* is a set of functions that loads and saves operational steps with a consistent naming structure. These need not be called from *pipeline.py*, since everything is taken care of in the appropriate operations (Code Snippet 3).

##### Operations—operations_functions.py

The file *operations_functions.py* is a set of functions that uses MNE-Python functions to apply the actual operations that are set with the pipeline script. These are set by the operations dictionary ([operations_to_apply], (Code Snippet 3))

##### Plotting—plot_functions.py

The file *plot_functions.py* is a set of convenience functions used for making a subset of possible plots. If [save_plots] is set to <True>, whatever is plotted will be saved in the given subject's figure directory (see [figures_path]). Since there are many variations on what plots one might want to create, I have included only very general plot functions that users can modify according to their own needs.

##### Paths

This sets the paths according to the structure of the downloadable data. <subjects_to_run> can be set to only a subset of the subjects (Code Snippet 2).

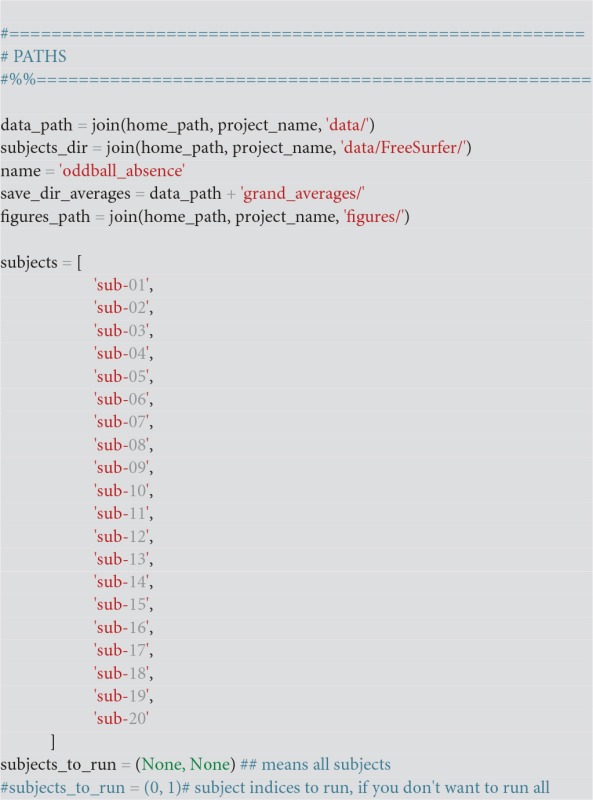



**Code Snippet 2**. Setting up the paths for structuring the data.

##### Operations

The operations block contains a dictionary with all the operations you can apply to the downloadable data. All values should be Boolean, meaning that they should be set to either True (1) or False (0). The appropriate operations for all values set to True will be applied to all subjects. The keys, e.g., “filter_raw,” correspond one-to-one in name with functions in *operations_functions* imported above (Code Snippet 1), except for keys that start with “plot_.” They correspond one-to-one with functions in *plot_functions* (Code Snippet 1). Make sure to run “populate_data_directory” before all the others. This will create all the necessary paths for the current analysis (Code Snippet 3). The operations are arranged in the order that they are most naturally performed, but since the output from each step is saved, one can jump into the analysis at any given point after a given step has been completed, if one wants to change some parameters. As an example of calculating the grand averages of the evoked fields and subsequently plotting the grand averages, the following keys need to be set to 1 (True): ***populate_data_directory; filter_raw; find_events; epoch_raw; run_ica; apply_ica; get_evokeds; grand_average_evokeds; plot_grand_average_evokeds*** or ***plot_grand_average_evokeds_butterfly***. Which functions are run by setting these keys will be shown below (Code Snippets 5–10 and 23). Another example is for running the MRI preprocessing necessary for creating a forward model. For this, the following keys need to be set to 1 (True): ***import_mri; segment_mri; apply_watershed; make_source_space; make_bem_solutions; create_forward_solution*** (Code Snippets 12–15 and 17–18). [In between a semi-manual transformation (**Figure 6**) bringing the MEG and MRI data into the same coordinate needs to be done, which can be made more precise using ***make_dense_scalp_surfaces*** (Code Snippet 16)].

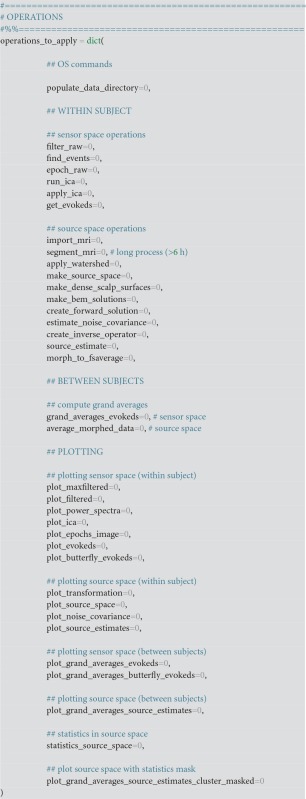



**Code Snippet 3**. Dictionary of operations that can be applied to the data. The value associated with each key (e.g., “filter_raw”) is a boolean, i.e., either True (1) or False (0).

##### Parameters

The variables here (Code Snippet 4) go into the functions as parameters that the comments above them associate them with, e.g., <lowpass> goes as a parameter into ***filter_raw*** (Code Snippet 5). Preset is a number of bad channels. <overwrite>, allows for making sure that overwriting is only done when explicitly requested, while <save_plots> determines whether plots are saved.

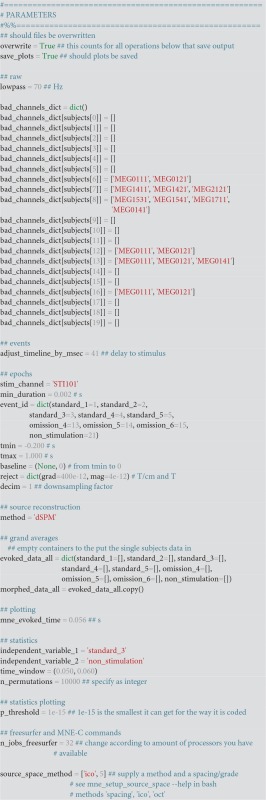



**Code Snippet 4**. Parameters that need to be set for the operations applied.

### Applying the operations

To reiterate: all functions mentioned below come from the script: *operations_functions.py*. They are dependent on the input/output-functions from *io_functions.py* that are always called from *operations_functions.py*. Which processing steps are run depend on what dictionary keys in <operations_to_apply> in *pipeline.py* are set to True. The user only needs to change *pipeline.py* to apply the functions described herein. The functions *operations_functions.py* and *io_functions.py* should not be changed, but more functions can be added for needs not covered in this protocol.

#### Preprocessing the MEG data

##### Dependencies

This part is only dependent on MNE-Python. All data plotted for single subjects is from subject *sub-01*.

##### MaxFilter

Since the MaxFilter software is proprietary software we do not expect everyone to have access to it, and thus the MaxFiltered data will be the starting point of the analysis from the MEG side.

##### Read MaxFiltered data and low-pass filter

Use ***filter_raw*** (Code Snippet 5) to read in the data and low-pass filter it according to [lowpass]. Three parameters, [name, save_dir, overwrite] occur for the first time here and are set by the corresponding variables <name, save_dir, overwrite> in *pipeline.py*. They determine the prepending name (oddball_absence) of the file to be saved, the path to which it should be saved, and finally whether it should be overwritten or not (True/False). Both the MaxFiltered and the low-pass filtered data can be plotted. This is done, respectively, with ***plot_maxfiltered*** and ***plot_filtered***. The power spectra for the raw data can be plotted with ***plot_power_spectra***. The effect of applying a low-pass filter is that it attenuates the contribution of frequencies above that cut-off while mostly preserving the contribution of frequencies below that cut-off. Since evoked responses are normally below frequencies of 30 Hz, the setting of the low-pass filter to 70 Hz should increase the signal-to-noise ratio by removing noise sources oscillating at frequencies >70 Hz. Signal-to-noise ratio might be improved even further by lowering the low-pass filter. This is left as an exercise to the user.

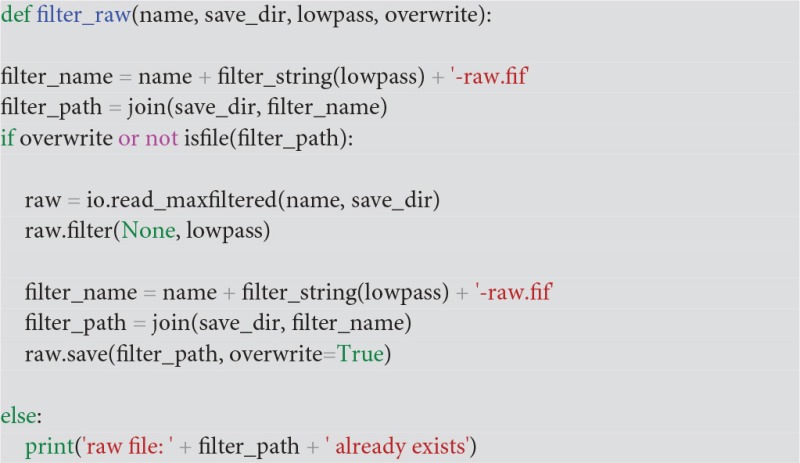



**Code Snippet 5**. The function for filtering the raw data.

##### Find events of interest and adjust timeline

Use ***find_events*** (Code Snippet 6) to find the events in the low-pass filtered data file and to adjust the events by the delay between the trigger and the actual event (the blowing up of the membrane). [stim_channel] and [min_duration] are used to set the stimulus channel and the minimum duration of an event in seconds, which are also their normal behaviours in MNE-Python. Events shorter than that are regarded as spurious and not included. [adjust_timeline_by_msec] is adjusting the events by the measured delay between the trigger value in the MEG recording and the actual blowing up of the membrane (41 ms).

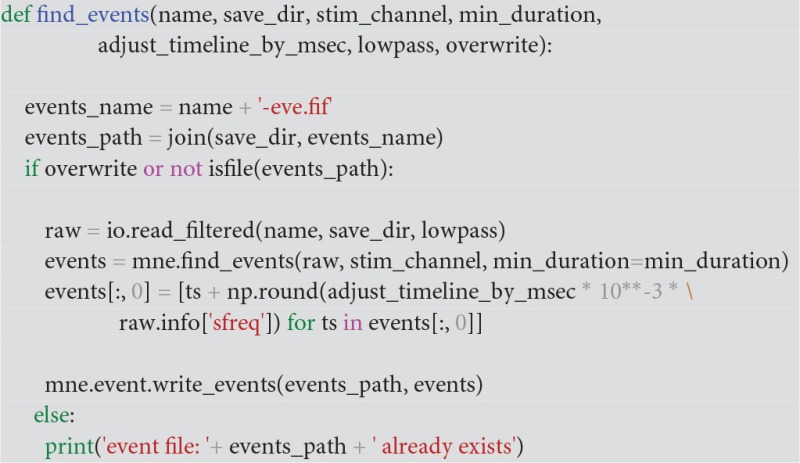



**Code Snippet 6**. The function for finding the events in the raw files. This function also adjusts the timeline for the events for the delay between the trigger and the actual event.

##### Epoch the raw data files

The parameters [event_id, tmin, tmax, baseline, reject, bad_channels, decim] all serve their normal purposes in MNE-Python. The [event_id] parameter is a dictionary indicating the names used for each event. In the code, this follows the naming in Table 1. The [tmin] and [tmax] parameters together define the time range (in seconds) around the triggers that make up each epoch, here chosen to be −0.200 and 1.000 s. After 1.000 s, one rarely sees evoked components, but this depends on one's paradigm. One should always check whether activity seems to return to baseline. In this case it does (**Figures 4, 9**). The [baseline] parameter indicates which part of the epoch, if any, should be used as a baseline. This demeans the whole epoch by the average magnetic field measured in the baseline time range. Here, the pre-stimulus time range, −0.200 to 0.000 s, is used, amounting to the assumption that there is no evoked activity of interest before the stimulation. This removes the offset response from each sensor and makes the evoked response amplitudes quantifiable relative to the pre-stimulus time range. It also removes the unwanted effects of slow drifts in the data (Gross et al., 2013), potentially leading to different offsets for each epoch. The [reject] parameter allows for automatically rejecting epochs where a given threshold value is exceeded, here chosen to be 4 pT for magnetometers and 400 pT/cm for gradiometers. These values are rejected since they are so high that they are not likely to arise due to neuronal activity. A list of subject-specific bad channels is passed to ***epochs_raw*** (Code Snippet 7) by [bad_channels], which have been filled in in <bad_channels_dict>. These have been assessed to contain very noisy data. When rejecting trials based on threshold values, it is always recommended to assess whether the rejection is due to a few bad channels. If this is so, it is advisable to just mark the relevant channel(s) as bad instead. For faster processing of the pipeline [decim] can be set to a higher value to downsample the data.

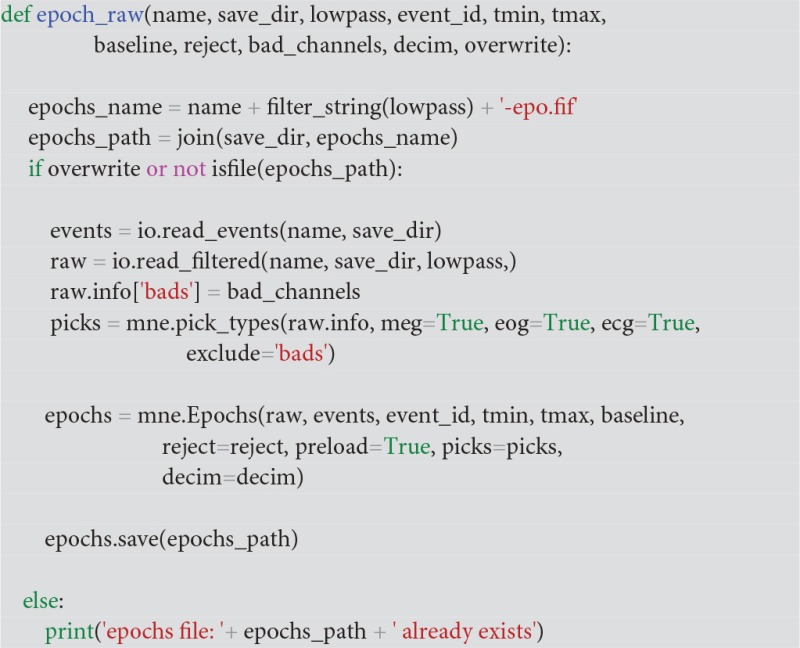



**Code Snippet 7**. The function for epoching the raw data, defining events, time before trigger, time after trigger, what to use as the baselining period, rejection threshold, which channels are bad and by which factor to decimate (downsample) the data.

##### Run independent component analysis (ICA)

**Figure 3:**
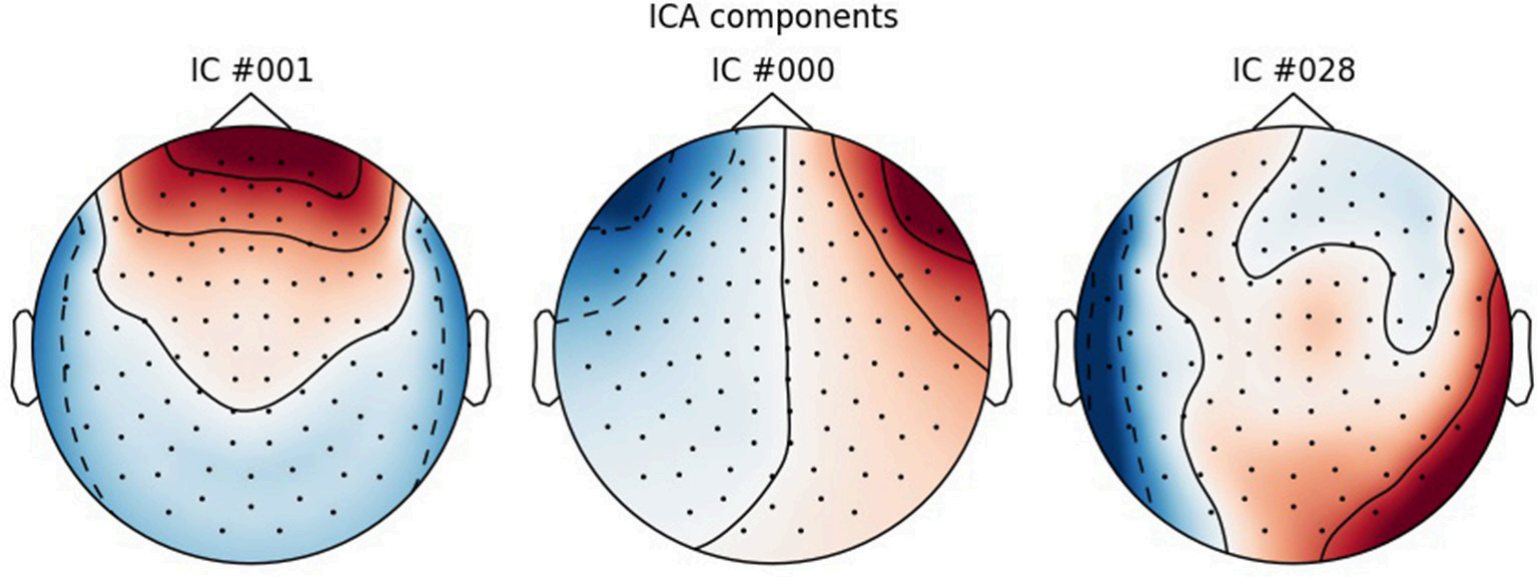
ICA components corresponding to eye blinks (ICA 000 and ICA 0001) and heart beats (ICA 028).

Use ***run_ica*** to estimate the independent components that explain the data the best, using the “fastica” algorithm (Hyvärinen, 1999). Epochs are then created that contain the electrooculographic- and electrocardiographic-related signals (eye blinks and heart beats). Next step is finding the indices for the components that correlate with eye blinks and heart beats. For the eye blinks this was done with Pearson correlation and for the heart beats this was done with the default method in MNE-Python, namely cross-trial phase statistics (Dammers et al., 2008). Finally, these components are removed from the ICA-solution, and the solution is saved. The removed components can be plotted with ***plot_ica*** (Figure 3). A particular issue that may arise when using ICA is that some components, say the heart beat component, may not be identifiable in all subjects. This would mean that it would not be possible to process all subjects in the same manner. There may be several reasons for this, e.g., the heart beat signal being only very weakly represented in the MEG data, as may happen for subjects where the distance between the heart and the head is great, i.e., tall subjects, or it may simply be that the recording is too noisy to faithfully record the electrocardiogram. The problem of having differently processed subjects is greatest in between-group studies where having different signal-to-noise ratios between groups may bias results. In within-group studies, the problem is thus less severe, since the decreased signal-to-noise ratio will apply to all conditions the given subject participated in, if ICA is run on all conditions collapsed, as is the case here. Alternative strategies for eye blinks and eye movements is to manually or automatically reject trials that contain eye blinks or excessive eye movements. To automatically reject trials that contain eye blinks or eye movements, one can add a key to the dictionary <reject> (Code Snippet 4) containing a threshold value for rejecting trials based on the electrooculogram. The process used here for ICA depends on automatic selection of components. These components should always be plotted to ascertain that they make sense, which can be done with ***plot_ica***. As artefact rejection always requires some subjective assessment, it is always useful to describe in some detail how these assessments were made. Following the suggestions for good practice by Gross et al. (2013) one should describe the ICA algorithm (fastica: Code Snippet 8), the input data to the algorithm (the epoched data: Code Snippet 8), the number of components estimated (sufficient number to explain at least 95% of the variance: Code Snippet 8), the number of components removed (three components: Figure 3) and the criteria for removing them (the aforementioned cross-trial phase statistics and Pearson correlation for heart beats and eye blinks respectively, which may be changed in ***ica.find_bads_eog*** and ***ica.find_bads_ecg***: Code Snippet 8). It should also be mentioned that one can use subjective assessment of whether components are likely to be related to eye blinks or heart beats (Andersen, this issue).

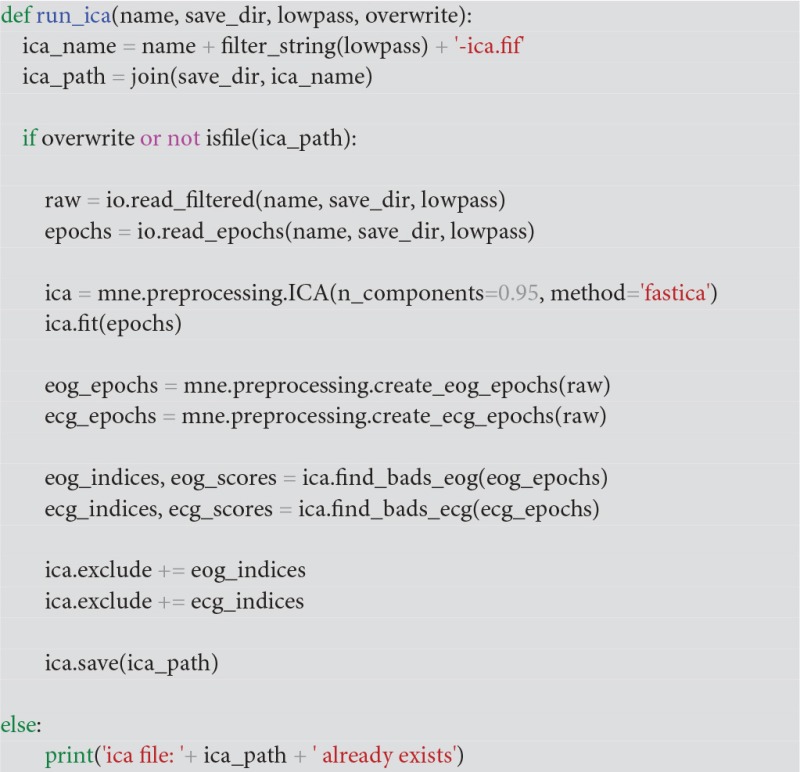



**Code Snippet 8**. The function for finding the independent components that most likely correspond to eye blinks, eye movements and heart beats.

##### Zero out eye- and heart-related components in the epoched data

Use ***apply_ica*** (Code Snippet 9) to zero out the components identified above to clean the data of eye- and heart-related activity.

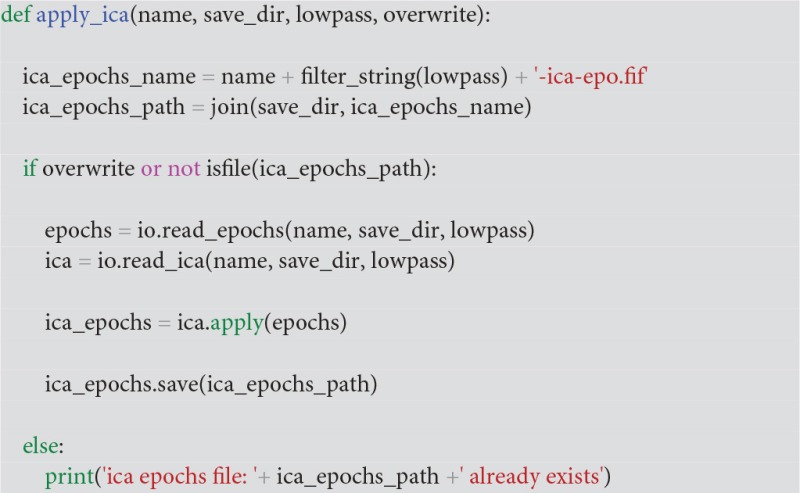



**Code Snippet 9**. The function for removing the eye blink, eye movement and heart beat components from the epoched data.

##### Event-related fields after relevant components have been removed

**Figure 4:**
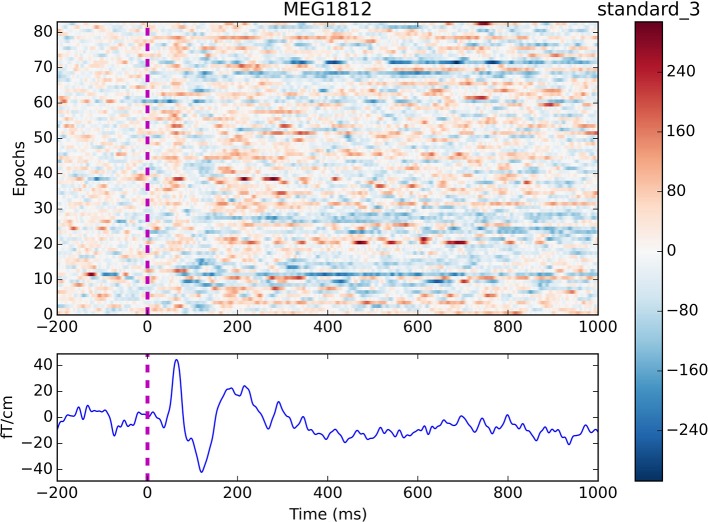
Epochs and event-related field for channel MEG 1812 for condition *standard 3*. The colouring indicates the field strength for each epoch. Two evoked responses can be seen after about 60 and 135 ms respectively.

Finally, the event-related fields are found for all the events of interest by looping through <epochs.event_id> and an averaged response is created for each event by calling ***get_evokeds*** (Code Snippet 10). The cleaned epochs can be plotted with ***plot_epochs_image*** (Figure 4). The event-related fields can be plotted with ***plot_evokeds*** and ***plot_butterfly_evokeds***.

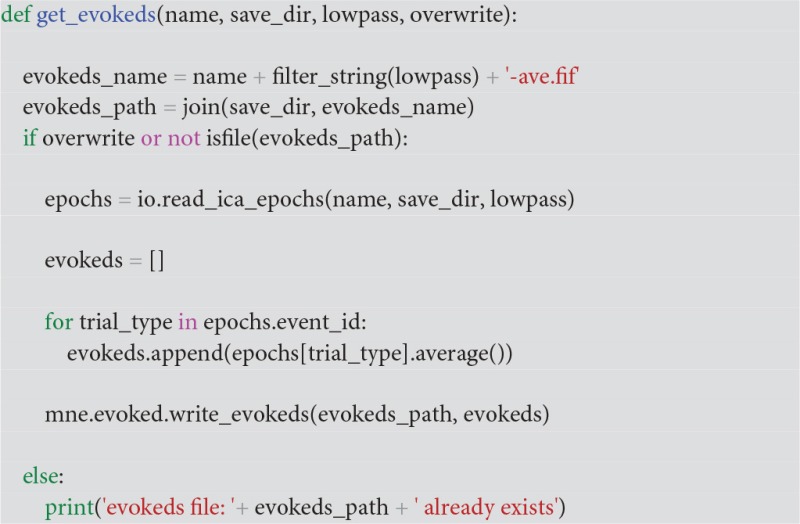



**Code Snippet 10**. The function for calculating the evoked responses based on the ICA-cleaned epochs.

##### Summary

This part of the code covered filtering of the data, finding events of interest, epoching of the data, estimating independent components, removing the eye- and heart-beat-related components and finally averaging the cleaned data. Expected evoked response can be seen after about 60 and 135 ms (Figure 4). The averages will be used for the subsequent source reconstruction of the data. To this end we need to preprocess the MRI data as well.

#### Preprocessing the MRI data

##### Dependencies

The python functions required for preprocessing the MRI data require FreeSurfer http://freesurfer.net/ and MNE-C http://martinos.org/mne. Both run exclusively on Linux and Mac platforms using the Bash language https://www.gnu.org/software/bash/. The plotting functions are based on MNE-Python. The function for creating high-resolution scalp surfaces also requires MATLAB. This is not strictly necessary for completing the source analysis, but is included since it aids in aligning the MEG and MRI coordinate systems. Due to concerns about subject anonymity, the original MRI data are not provided. The “bem” folder for each subject in <subjects_dir> is provided though, as this information is judged non-sensitive. The python functions, ***import_mri***, ***segment_mri*** and ***apply_watershed*** (Code Snippets 12–14) can thus not be applied to the data, but they are included such that users can these in their own experiments. They cover reading in dicom files, segmenting the brain and delineating the surface between brain, skull and skin. The functions below (Code Snippets 12–17) all use the local function ***run_process_and_write_output*** to call the commands in Bash and print the output of the operations in the Python console (Code Snippet 11).

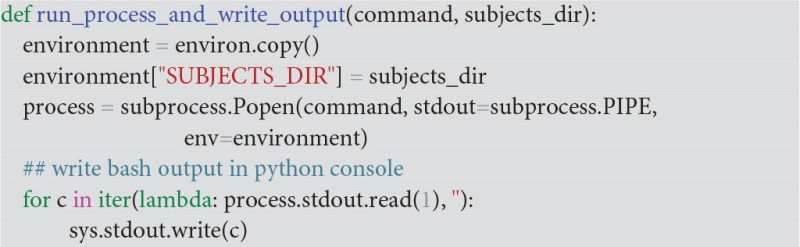



**Code Snippet 11**. The local function used for calling Bash commands, setting the subjects_dir, and printing the outputs of the Bash commands in the Python console.

##### Read in dicom files

Use Code Snippet 12 to read in the MR1s. This creates a subject folder in the SUBJECTS_DIR directory required by FreeSurfer.

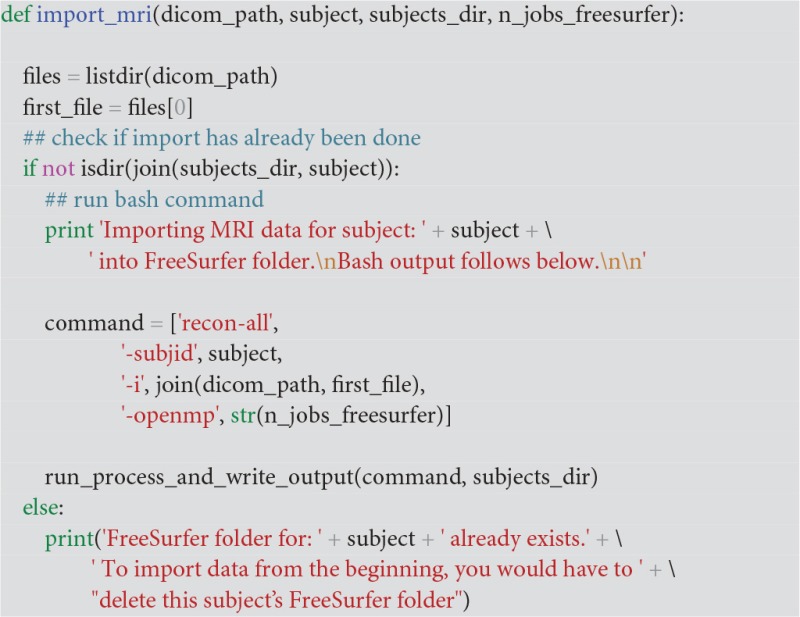



**Code Snippet 12**. Code for importing the dicom files into the FreeSurfer folder, which FreeSurfer requires.

##### Segment the MRI

Use Code Snippet 13 to do the full segmentation of the brain into its constituent parts using FreeSurfer. [openmp] sets the number of processors that FreeSurfer will use. This is a very lengthy process and takes between ~6–24 h for each subject depending on processing power.

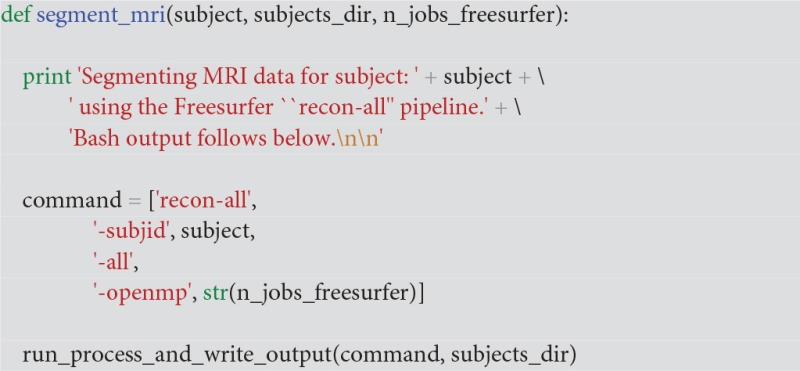



**Code Snippet 13**. Code for doing a full FreeSurfer segmentation (a very lengthy process).

##### Create boundaries with the boundary element method (BEM) using the watershed algorithm

Use Code Snippet 14 to create surfaces for the inner skull, the outer skin, the outer skull and the brain surface with an MNE-C command, which uses FreeSurfer code. Copies of the watershed files are created in the bem-folder for each subject since this is where MNE-C expects to find them.

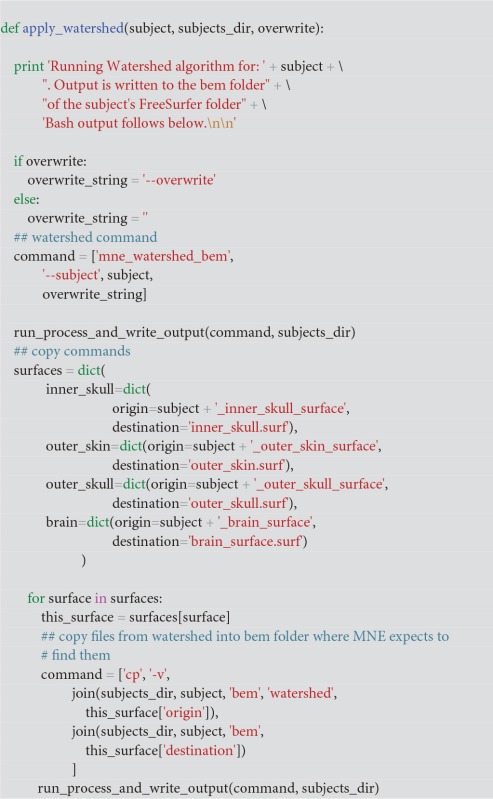



**Code Snippet 14**. Code for creating the boundary elements necessary for defining the volume conductor.

##### Make source spaces

Use Code Snippet 15 to create a source space that is restricted to the cortex with ~10,000 sources modelled per hemisphere as equivalent current dipoles normal to the cortical surface.

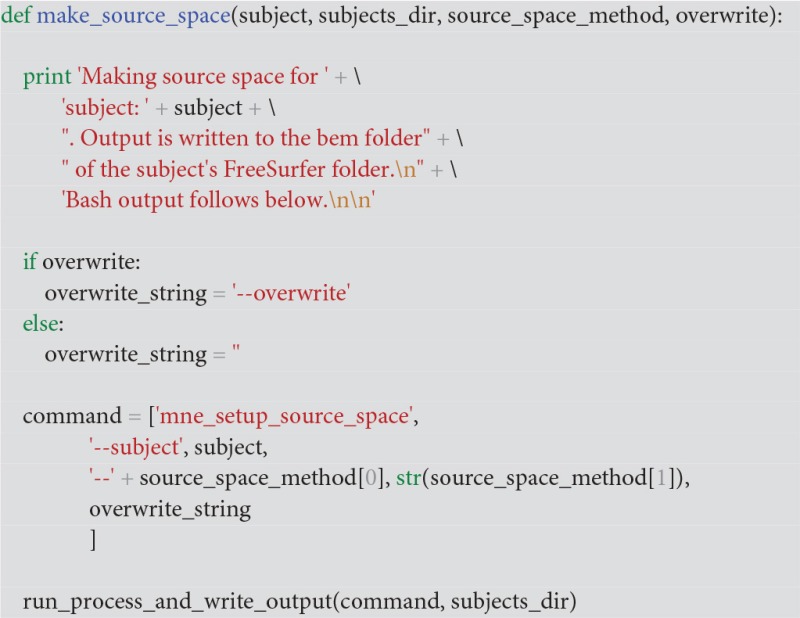



**Code Snippet 15**. Code for making the source space.

**Figure 5:**
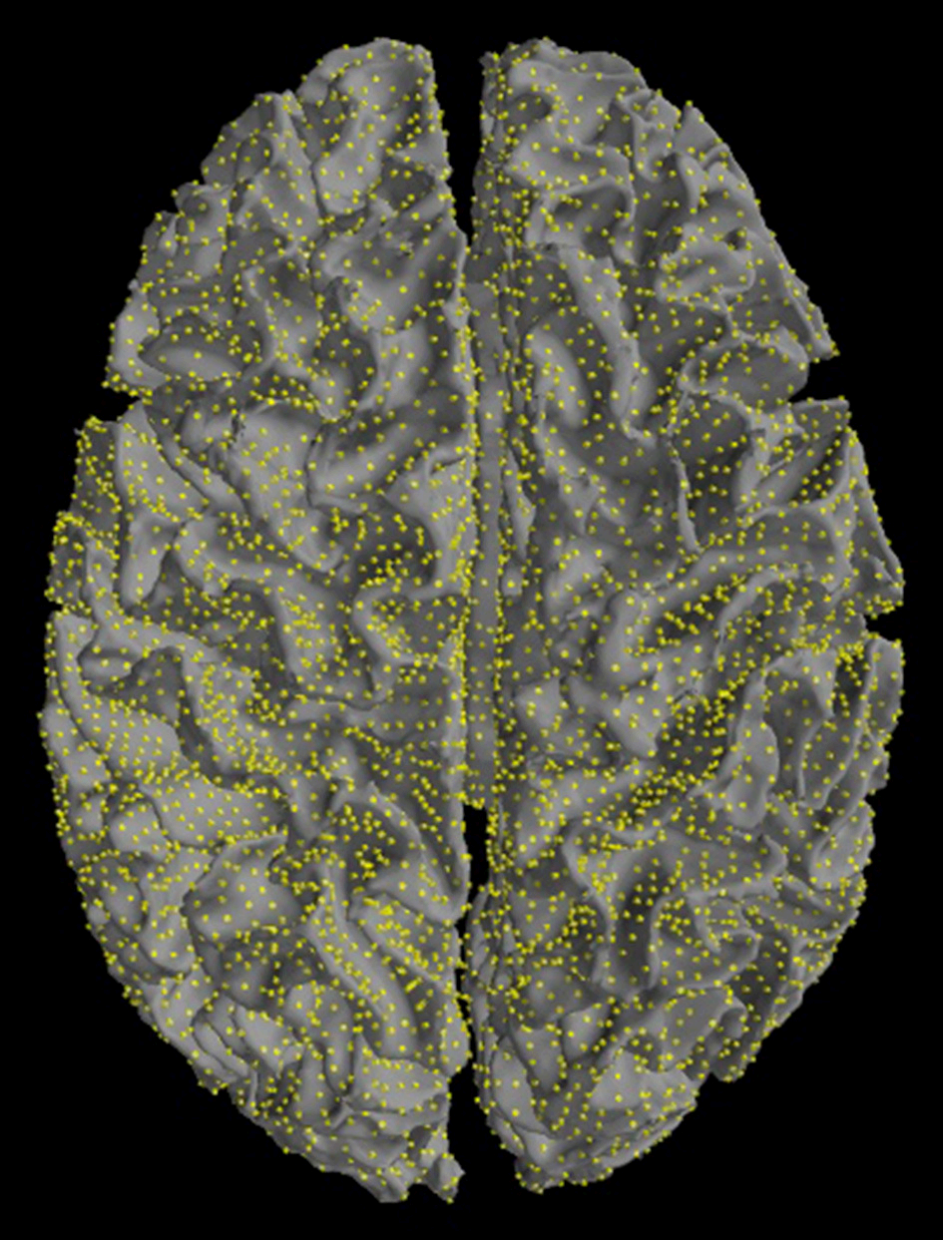
Source space. Sources are restricted to the cortex. Yellow dots mark equivalent current dipoles on the cortical surface.

The source space can be plotted with ***plot_source_space*** (Figure 5).

##### Make scalp surfaces

Use Code Snippet 16 to make high-resolution scalp surfaces for each subject. This eases the co-registration since it makes it easier to identify the fiducials, nasion and left and right pre-auricular points. The MNE-C code here is dependent on MATLAB, but the high-resolution scalp surfaces are not strictly necessary for the completing the analysis. Their purpose is to ease the co-registration of the MEG and MRI data.

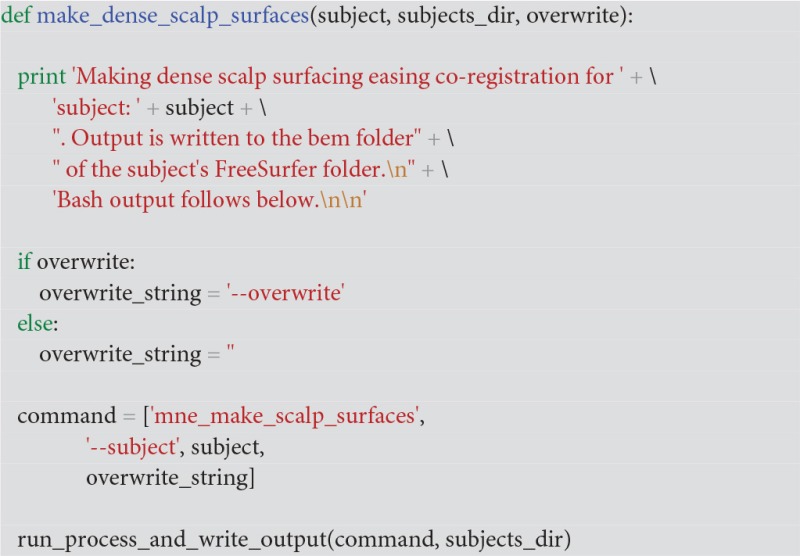



**Code Snippet 16**. Code for making high-resolution scalp surfaces.

##### Create solutions for the BEMs

Use Code Snippet 17 to create a volume conductor model describing how the magnetic fields spread throughout the conductor (the head). [homog] makes a single-compartment model (sensible for MEG). [surf] instructs MNE-C to use the surfaces created with the watershed algorithm. [ico] determines the downsampling of the surface. [ico, 4] results in ~10,000 sources for the two hemispheres.

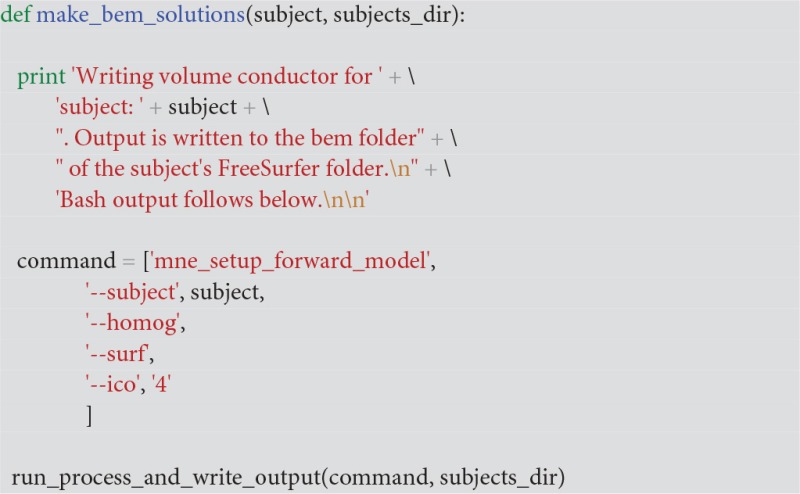



**Code Snippet 17**. Code for making the BEM-solutions, that is the volume conductor.

#### Source reconstruction of time courses

##### Co-registration

**Figure 6:**
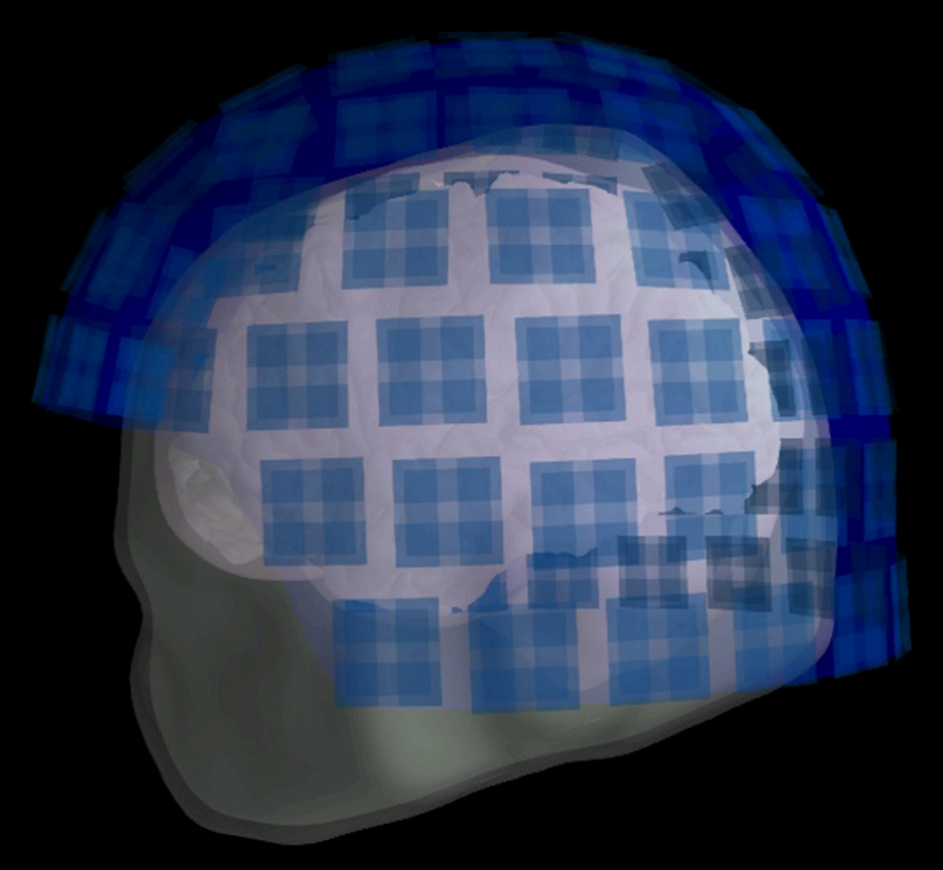
Transformation. The positions of the head, skull, brain, and helmet sensors after the transformation.

Call the function ***mne.gui.coregistration*** directly from a Python environment to co-register the MEG data to the MRI data. Fiducials used are the nasion and the left and right pre-auricular points. The scalp surfaces made above should make it easier to identify these fiducials. When these have been set, load a file that has the extra head shape digitization points and lock the fiducials. Then fit the head shape, and if the fit looks good save the transformation file as “oddball_absence_dense-trans.fif” in the same folder where all other MEG data files are saved. The resulting transformation can be plotted with ***plot_transformation*** (Figure 6). Note that a transformation with this name has already been supplied, such that the analysis can be replicated faithfully.

##### Create forward model

Use ***create_forward_solution*** (Code Snippet 18) to create the forward model for the source reconstructions. This contains the source space, the volume conductor model, the transformation between the MEG and MRI coordinate systems and information about the channels in the data. The forward model is linking the source model (where sources are and how sources are oriented) to the sensors in the recording system. The volume conductor models how the magnetic field spreads from the sources, here we modelled them as spreading homogeneously (Code Snippet 17), to the sensors, whose positions are stored in the information field of the raw data. The co-registration is necessary to make sure that the MRI data and the MEG sensors are in the same coordinate space. In physical units, the forward model contains the magnetic field/gradient estimates for each sensor for each source given a unit-activation of the source (1 nAm).

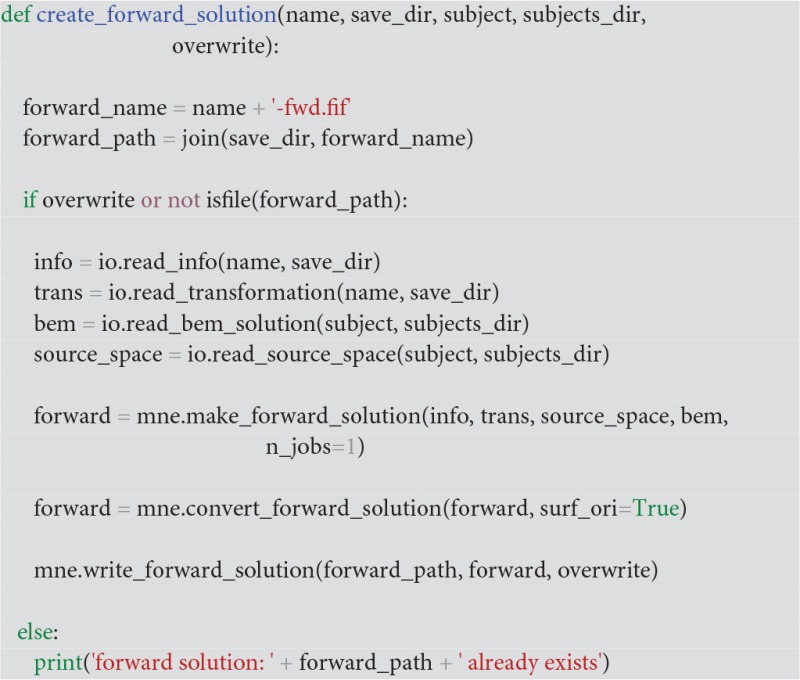



**Code Snippet 18**. Function for creating the forward solution (also known as the lead field). This is created from the BEM-solution (the volume conductor), the channel info about the sensor positions, the coordinate transformation between the MEG and the MRI data and the source space defining where sources are.

##### Estimate noise covariance

**Figure 7:**
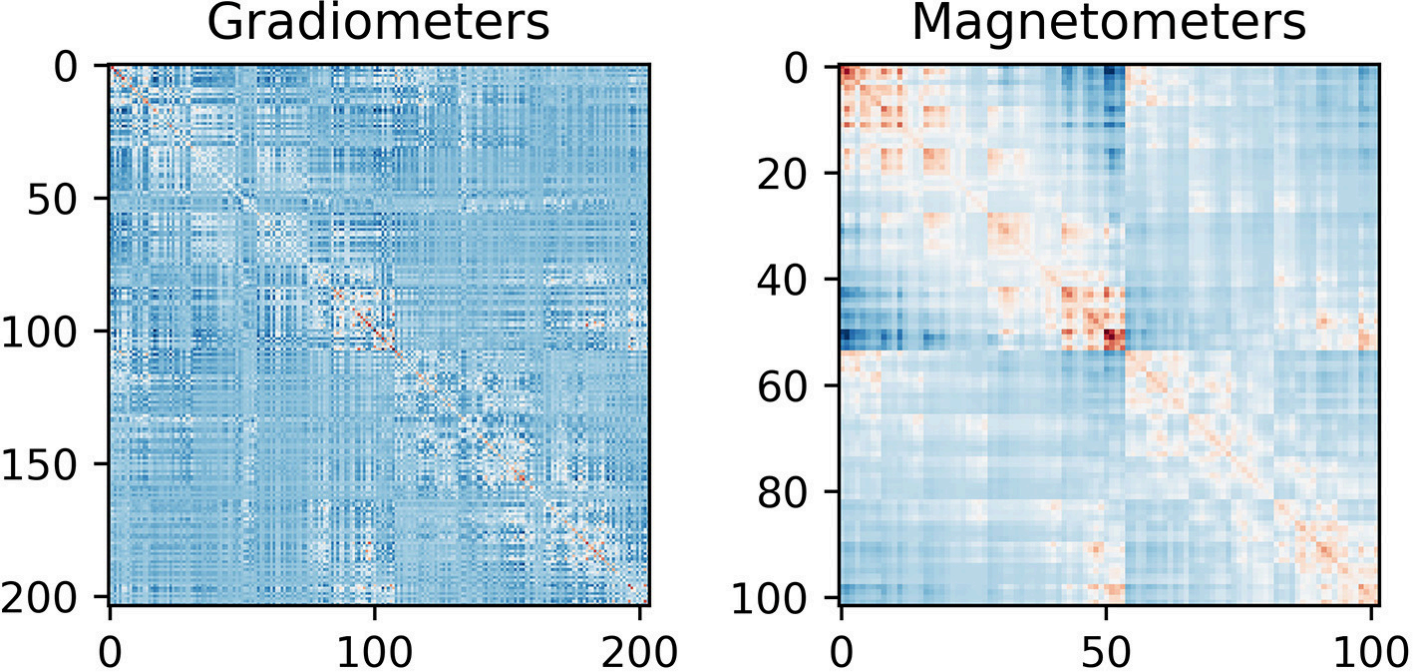
Noise covariance matrices. As can be seen the covariance between magnetometers is greater than between gradiometers. This can be explained by magnetometers being more sensitive to far away sources than gradiometers are.

Use ***estimate_noise_covariance*** (Code Snippet 19) to estimate the noise covariance and regularize it. The noise covariance serves as an estimate of the noise in the data, which is necessary for MNE-like solutions. Regularization is done since the smallest eigenvalues of the noise covariance matrix might be inaccurate, thus giving rise to errors in the source estimates. The noise covariance matrix can be plotted with ***plot_noise_covariance*** (Figure 7). To investigate more thoroughly whether regularization is necessary, one can set the parameter [method] in ***mne.compute.covariance*** to “auto” to test which of several ways of estimating the covariance is optimal (Engemann and Gramfort, 2015). This is a lengthy process though (>12 h) on a modern computer, but will give estimates, among other things, on whether regularization would improve the estimate of the noise in the data. It is possible though just to compare whether or not regularization should be applied to the noise covariance matrix estimated by using the trials, by just comparing the “diagonal_fixed” and “empirical” methods using ***mne.compute.covariance***, which is much faster (on the scale of minutes), This also allows for comparing different degrees of regularization. Including the regularization done here allowed for better noise covariance matrices for all subjects when compared to not including regularization, according to the approach of Engemann and Gramfort (2015).

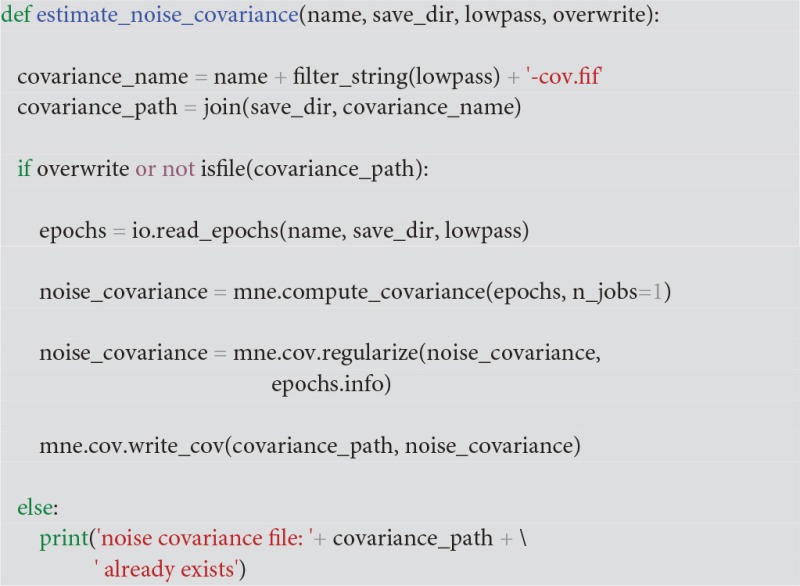



**Code Snippet 19**. Function for estimating the noise covariance in the MEG data.

##### Create the inverse operator

The final step before estimating source activity is to create an inverse operator, which contains the info about the MEG-recordings, the estimated noise, the source reconstruction method used and the forward model. This is done with ***create_inverse_operator*** (Code Snippet 20).

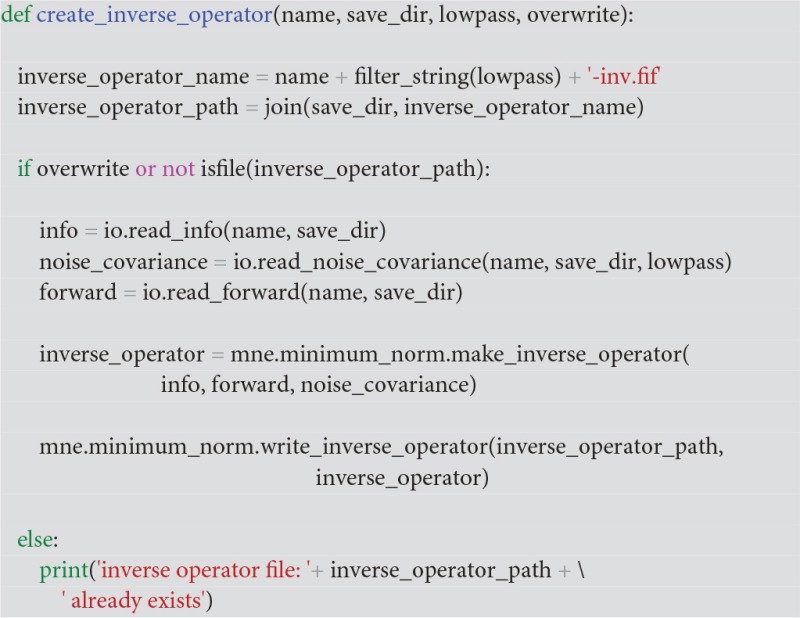



**Code Snippet 20**. Function for creating the inverse operator that defines what inverse solution should be applied.

##### Estimating the source time courses

Finally, we estimate the source time courses. [method] is set in the parameter selection. *dSPM* is a depth-weighted minimum source estimate (Dale et al., 2000), *MNE* is the classical algorithm described by Hämäläinen and Ilmoniemi (1994) and sLORETA is described by Pascual-Marqui (2002). A source time course (stc-file) is created for each condition. This is done with ***source_estimate*** (Code Snippet 21). Here, dSPM is chosen as the [method] parameter since it is known to reduce the bias that MNE has toward superficial cortical areas.

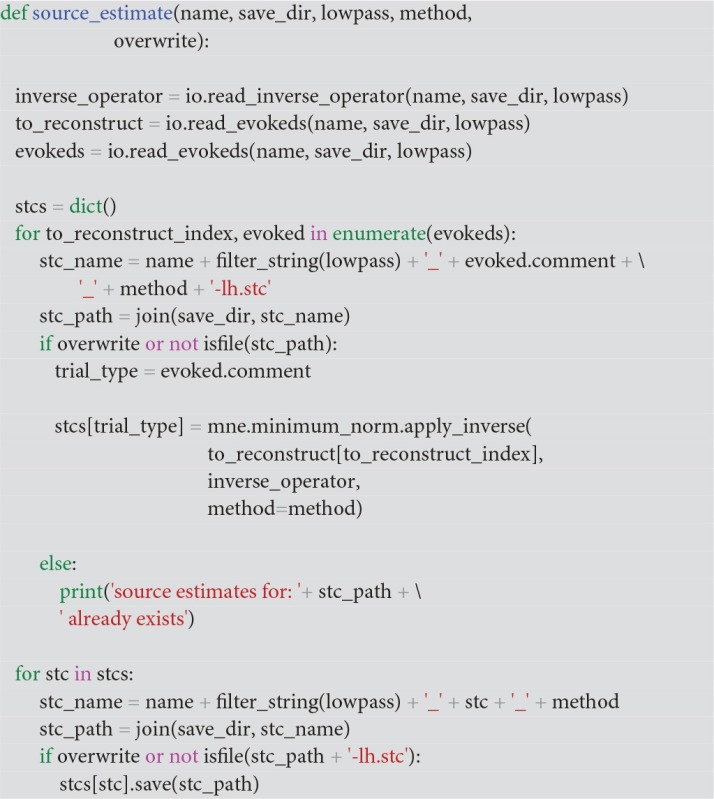



**Code Snippet 21**. Function for doing the actual minimum norm estimate source reconstruction.

**Figure 8:**
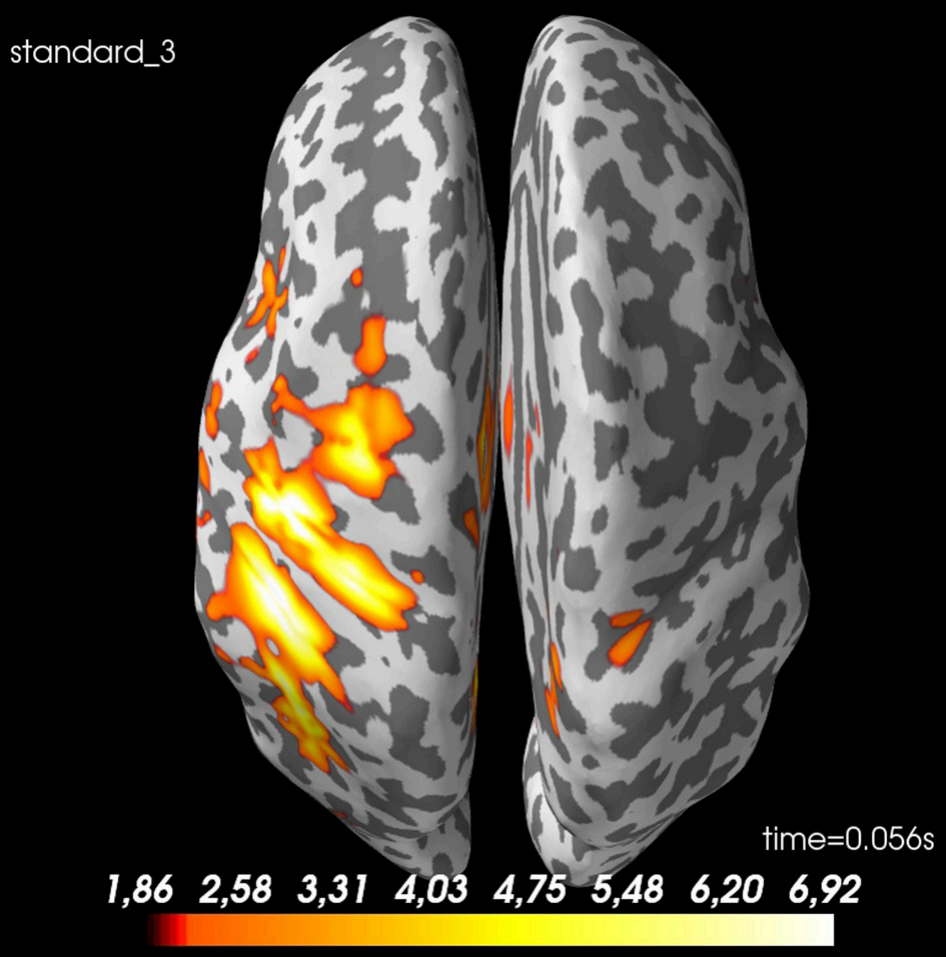
Spatial distribution of neural activity at 56 ms for *standard 3* for *sub-01*: There is some spread, but there is a clear activation of the contralateral sensory cortex. Values are dSPM-values. These are current estimates normalized with the noise-covariance. The cortex is shown inflated with gyri darker than sulci.

The spatial source distribution for a given time point can be plotted with ***plot_source_estimates*** (Figure 8). <mne_evoked_time> in *pipeline.py* can be set to control which time point is plotted.

##### Morph to a common template

Use ***morph_data_to_fsaverage*** (Code Snippet 22) to make a meaningful estimate across subjects, by morphing the data from each individual subject to a common template brain. In this case, the fsaverage brain from FreeSurfer is used (This requires the fsaverage brain to be in $SUBJECTS_DIR). [method] can be “dSPM,” “MNE,” or “sLORETA.”

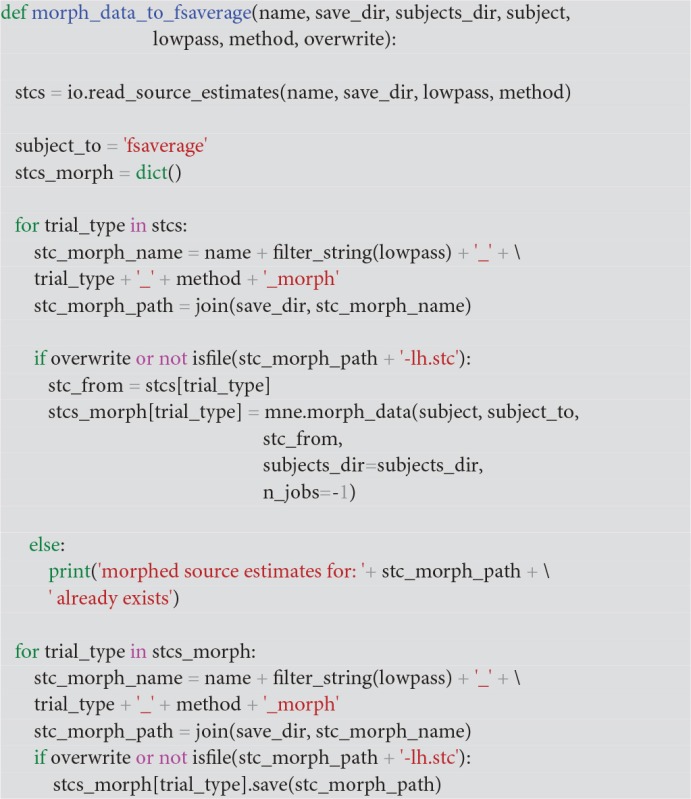



**Code Snippet 22**. Function for making morph maps that define how individual subject source reconstructions can be mapped onto a common template that allows for comparisons between subjects.

##### Summary

Now we have estimated source time courses for all the individual subjects. The next step is to meaningfully make a group estimate across subjects. The activity for our example subject (sub-01) can be localized to the somatosensory cortex (Figure 8) as was expected.

#### Between subjects analyses

##### Dependencies

This part is only dependent on MNE-Python.

##### Sensor space

**Figure 9:**
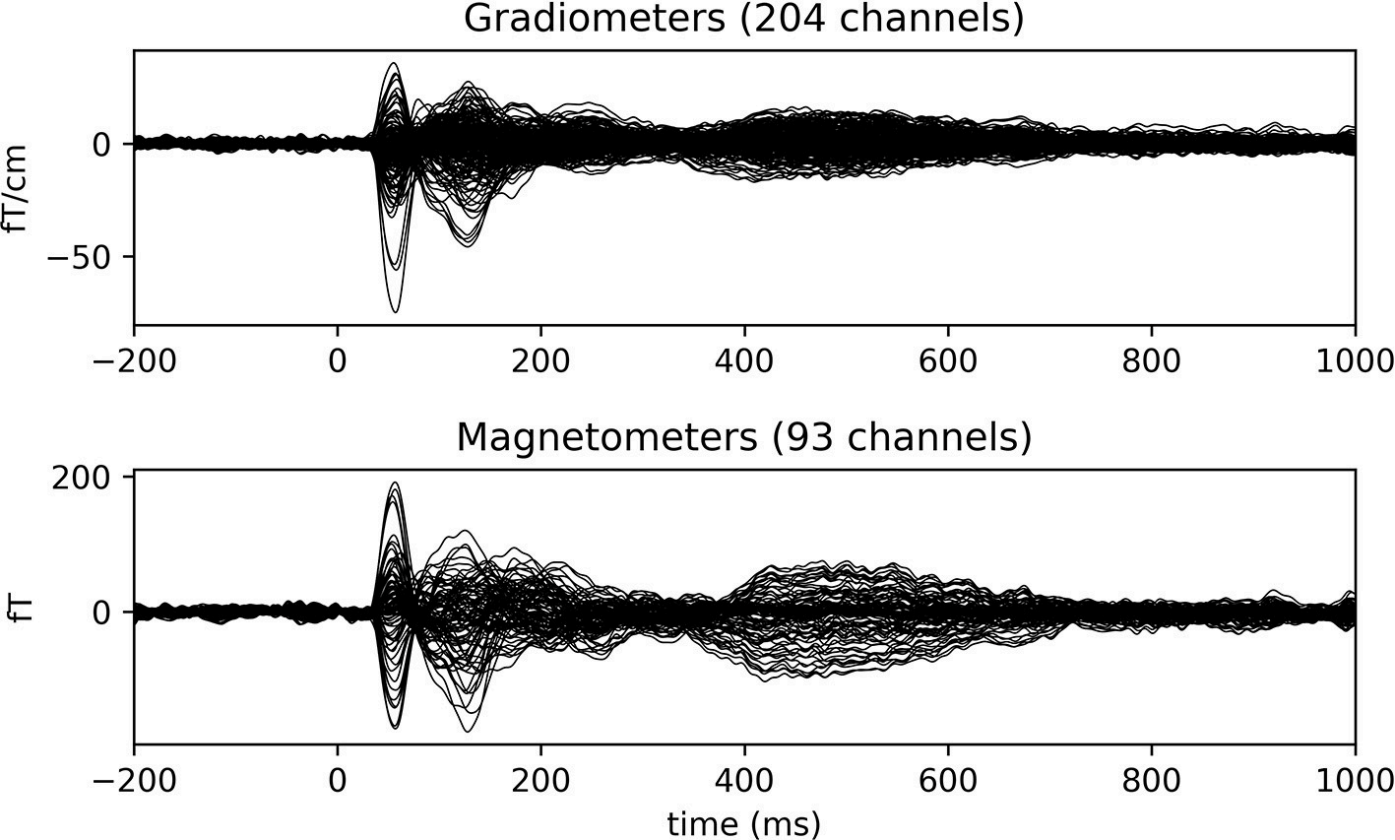
Grand average butterfly plot for *standard 3* showcasing the SI (56 ms) and SII (135 ms) components.

With the function ***grand_average_evokeds*** (Code Snippet 23), the grand average in sensor space for each condition is calculated and saved. Grand averages can be plotted with ***plot_grand_averages_evokeds*** and ***plot_grand_averages_butterfly_evokeds*** (Figure 9). Note that these may not be easy to interpret since the relative positions between a given subject's head and the MEG sensors will differ from the relative positions between any other subject's head and the MEG sensors. The early and the late responses are picked up however (Figure 9).

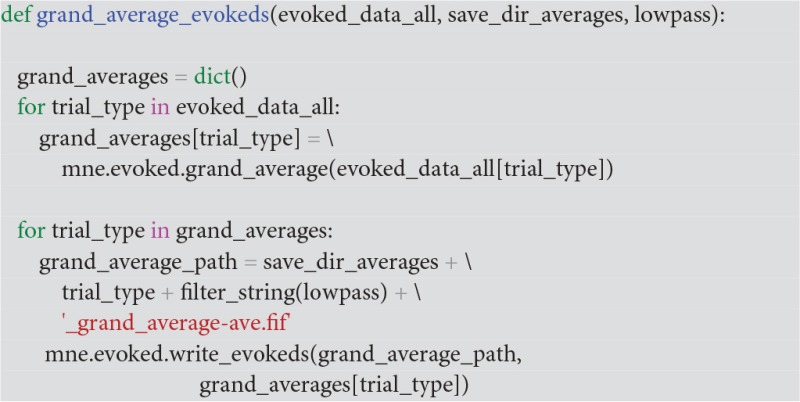



**Code Snippet 23**. Function for calculating grand averages across the evokeds of individual subjects.

##### Source space

**Figure 10:**
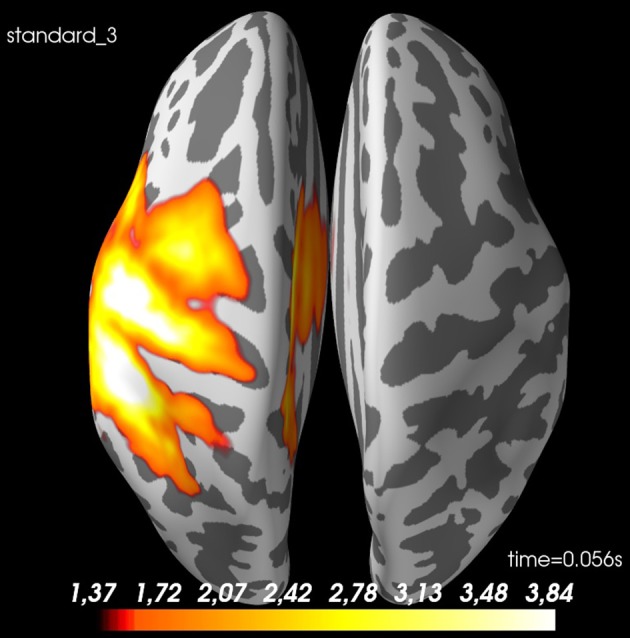
Spatial distribution of neural activity at 56 ms for grand average of *standard 3*: There is some spread, but there is a clear activation of the contralateral sensory cortex. Values are dSPM-values. These are current estimates normalized with the noise-covariance. The cortex is shown inflated with gyri darker than sulci.

With the function ***average_morphed_data*** (Code Snippet 24), the grand average in source space over the morphed source time courses for each condition is calculated and saved. [method] can be “dSPM,” “MNE,” or “LORETA.” The grand averages for the source space can be plotted with ***plot_grand_averages_source_estimates*** (Figure 10). <mne_evoked_time> needs to be set.

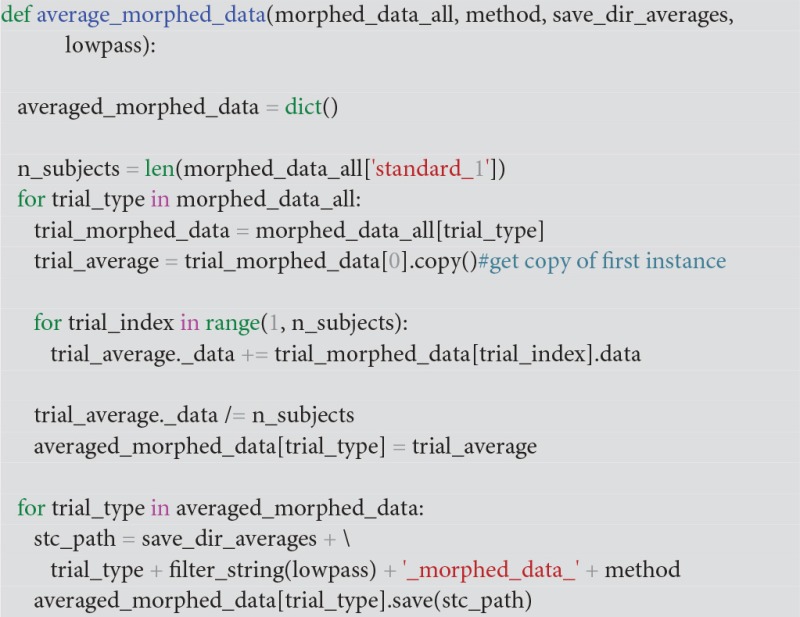



**Code Snippet 24**. Function for calculating the grand average across all individual morphed subject source reconstructions.

### Statistical analyses

This part is only dependent on MNE-Python.

With the function ***statistics_source_space*** (Code Snippet 25), different statistical null hypotheses can be tested.

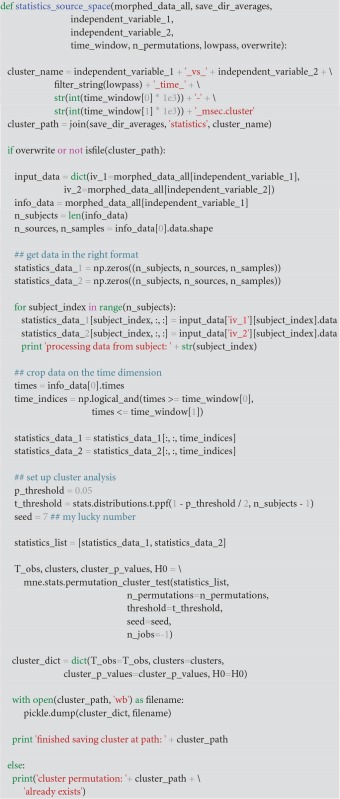



**Code Snippet 25**. Function for doing cluster statistics in source space.

**Figure 11:**
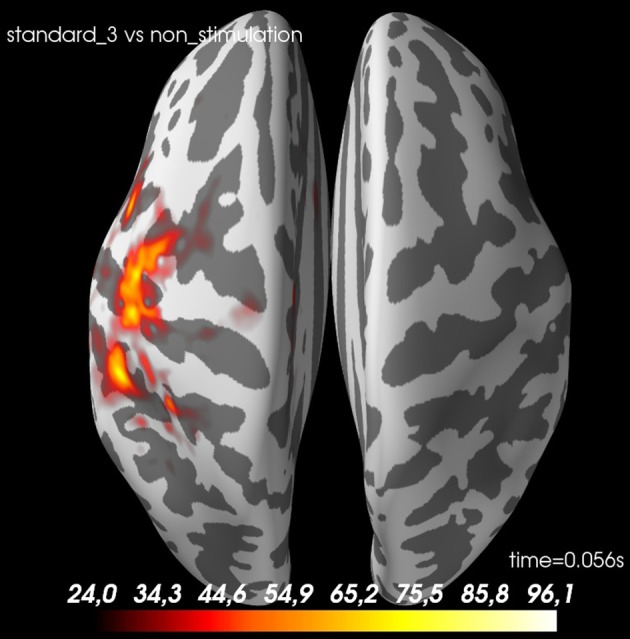
A *t*-value map for *standard 3* vs. *non-stimulation* at 56 ms. The cortex is shown inflated with gyri darker than sulci.

<independent_variable_1>, <independent_variable_2>, <time_window> and <n_permutations> should all be set. With plot_grand_averages_source_estimates_cluster_masked (Figure 11) the t-masked grand average source estimates can be plotted. <p_threshold> should be set. This function can be changed such that any other function in the mne.stats module is used.

## Summary

This protocol allows for all steps of conducting a MEG group study aiming to provide evidence for a significant effect of one experimental condition compared to another experimental condition using Minimum Norm Estimates of MEG data. We found as expected that stimulation of the finger elicited more activity in the contralateral somatosensory cortex than when no such stimulation occurred.

## Discussion

The presented pipeline allows for covering all steps involved in an MNE-Python pipeline focusing on evoked responses and the localization of their neural origin. Furthermore, it also supplies a very flexible framework that users should be able to extend to meet any further needs that the user may have. Facilitating other MNE-Python functions not showcased here across groups of subjects can be attained by emulating the style of defining functions presented here. If one is interested in estimating induced responses, one can use the functions in the ***mne.time_frequency*** module. The neural origin of induced responses are often localized with beamformer solutions (Gross et al., 2001), which can also be performed with MNE-Python using the ***mne.beamformer*** module. Both these can be extended by a user with some programming experience.

The present pipeline is all contained within a single pipeline script and three function scripts containing the functions called from the pipeline. Another way of organizing one's data is to creating batches using build systems like GNU Make (https://www.gnu.org/software/make/) (Stallman et al., 2002), luigi (https://luigi.readthedocs.io/en/stable/), doit (http://pydoit.org/).

## Author contributions

The author confirms being the sole contributor of this work and approved it for publication.

### Conflict of interest statement

The author declares that the research was conducted in the absence of any commercial or financial relationships that could be construed as a potential conflict of interest.
